# Genetic inhibition of CRMP2 phosphorylation at serine 522 promotes axonal regeneration after optic nerve injury

**DOI:** 10.1038/s41598-019-43658-w

**Published:** 2019-05-10

**Authors:** Shunsuke Kondo, Kazuya Takahashi, Yuki Kinoshita, Jun Nagai, Shuji Wakatsuki, Toshiyuki Araki, Yoshio Goshima, Toshio Ohshima

**Affiliations:** 1 Department of Life Science and Medical Bio-Science, Waseda University, Shinjuku-ku, Tokyo 162-8480 Japan; 2 Research Fellow of Japan Society for the Promotion of Science, Chiyoda-ku, Tokyo Japan; 3 Department of Peripheral Nervous System Research, National Institute of Neuroscience, National Center of Neurology and Psychiatry, 4-11 Ogawa-higashi, Kodaira, Tokyo 187-8502 Japan; 4 Department of Molecular Pharmacology and Neurobiology, Yokohama City University Graduate School of Medicine, Yokohama, 236-0004 Japan; 5 Institute for Advanced Research of Biosystem Dynamics, Waseda Research Institute for Science and Engineering, Waseda University, Shinjuku-ku, Tokyo Japan

**Keywords:** Phosphorylation, Regeneration and repair in the nervous system

## Abstract

Axonal degeneration occurs in various neurological diseases and traumatic nerve injury, and axonal regeneration is restricted by inhibitory factors in the central nervous system. Cyclin-dependent kinase 5 and glycogen synthase kinase 3β (GSK3β) are activated by one of those inhibitors, and collapsin response mediator protein 2 (CRMP2) is phosphorylated by both kinases. We previously developed a CRMP2 knock-in (CRMP2 KI) mouse line, in which CRMP2 phosphorylation at Ser 522 is inhibited. Because CRMP2 KI mice showed promotion of axonal regeneration after spinal cord injury, we hypothesized that CRMP2 KI mice would show higher axonal regeneration after optic nerve injury. In this study, we first show that depolymerization of microtubules after optic nerve crush (ONC) injury was suppressed in CRMP2 KI mice. Loss of retinal ganglia cells was also reduced after ONC. We found that protein level of GAP43, a marker of regenerative axons, was higher in the optic nerve from CRMP2KI than that from wild type 4 weeks after of ONC. We further observed increased numbers of axons labeled by tracer in the optic nerve after ONC in CRMP2 KI mice. These results suggest that inhibition of phosphorylation of CRMP2 suppresses axonal degeneration and promotes axonal regeneration after optic nerve injury.

## Introduction

In the mature mammalian central nervous system (CNS), injured axons have a limited capacity to regrow. Therefore, it is difficult for axons to regenerate and recover their function when faced with degenerative disorders of CNS. Currently, no effective method exists for completely repairing the CNS after injury or degeneration. However, previous studies have discovered potential mechanisms that inhibit CNS axonal regeneration, such as the deficiency of intrinsic regrowth capacity in mature CNS neurons^1^, absence of external growth stimulating factor^2,3^, and/or presence of external inhibitory factors^4,5^. Addressing these factors could lead to the development of novel CNS disease treatments.

The optic nerve is a part of the CNS and transmits visual information from the retina to the brain. Optic neuropathies can cause blindness or loss of visual function. Optic neuropathies, such as glaucoma and traumatic injury, commonly involve the eventual death of retinal ganglion cells (RGCs), the projection neurons of the eye^6^. RGC death generally occurs through apoptosis, which is caused by RGC axotomy^7,8^. Optic nerve crush (ONC) is one of the methods of surgical RGC axonal injury causing axonal degeneration and apoptotic RGC death.

Collapsin response mediator protein 2 (CRMP2), a CRMP family molecule, was identified as a mediator in Sema3A signaling in CNS development^9^. One of the mechanisms of CRMP2 is to stabilize microtubules and promote polymerization by binding to tubulin dimers^10,11^. In the Sema3A-induced axonal growth cone collapse signaling pathway, cyclin-dependent kinase 5 (Cdk5) inactivates CRMP2 through phosphorylation at Ser522, which primes for GSK3β phosphorylation at Ser518, Thr514, and Thr509, leading to a destabilization of microtubules^12,13^. GSK3β activation and GSK3β-mediated CRMP2-phosphorylation was reported in optic nerve after injury^14^. The inhibition of this GSK3β-CRMP2 pathway provides protection against axonal degeneration^14^.

To examine the roles of phosphorylation of CRMP2 after optic nerve crush (ONC), we used CRMP2 knock-in (CRMP2 KI) mice, in which the Ser522 residue of CRMP2 was replaced with Ala, leading to the elimination of phosphorylation at Ser522 and the subsequent phosphorylation at the Ser518, Thr514, and Thr509 of CRMP2^15^. Our previous study showed promoted axonal regeneration in CRMP2 KI mice after spinal cord injury^16^. In the present study, to investigate the role of CRMP2 in axonal stabilization and regeneration *in vivo*, we compared degeneration and regeneration of the optic nerve between wildtype and CRMP2 KI mice after optic nerve injury. Our experimental data indicate that inhibition of CRMP2 phosphorylation promotes optic nerve regeneration after injury.

## Materials and Methods

### Animals and surgical procedures

The mice used in the experiments were housed in accordance with the technical protocols for animal experiments approved by the Institutional Animal Care and Use Committee at Waseda University (2015-A-023, 2016-A006, 2017-A027). Wild-type and CRMP2 S522A mutant (CRMP2 KI) mice were generated as previously described^15^. All mice were adult males aged between 10 and 16 weeks (wk) at the time of operation. Optic nerve crush (ONC) were introduced at the left optic nerve alone, while the right side was not injured and served as a control according to the method previously described^17^. Briefly, mice were anesthetized with an intraperitoneal injection of Avertin (400 µl; 12.5 mg/ml), and the left optic nerve was exposed and crushed for 5 s with a reverse action tweezers (P-652, Hozan, Osaka, Japan) at a site about 1 mm behind the globe^17^. Axonal degradation was confirmed using yellow fluorescent protein-H (YFP-H) mice (Sup. Fig. 1)^18^, that were previously used for quantitative analysis of the grade of axonal degradation resulting from ONC^19,20^.

### Immunostaining

Mice were perfused intracardially with 4% paraformaldehyde (PFA) in 0.1% phosphate buffer (pH 7.4) 1, 2 or 4 wk after ONC. Retinas were subsequently dissected. Four slits at even intervals were made in each retina so that they laid flat. They were fixed in 4% PFA for 24 h, washed in TBS for 10 min, placed in methanol for 10 min, washed in Tris-buffered saline (TBS) for 10 min, incubated for 30 min in blocking solution, 3% house serum in TBS with 0.1% Tween 20 (TBST), and incubated overnight with the primary antibodies at 4 °C. After washing in TBST for 30 min three times, the Alexa-Fluor secondary antibodies were applied at room temperature (RT) for 2 h. Retinas were then washed in TBST for 30 min 2 times and placed in TBS for 30 min enclosed with 50% glycerol. For the immunostaining of RGCs, anti-RNA-binding protein with multiple splicing (RBPMS) antibody (1:500, rabbit polyclonal antibody PA5-31231, Invitrogen, CA) was used as the primary antibody. RBPMS was used as a specific marker for RGCs^21^. The number of RGCs in counted areas of each sample was 12 places per single retina and one place was 0.31 × 0.31 mm. There are 3 points, 0.5, 1.0, and 1.5 mm, from the optic disk, in 4 areas.

Optic nerves were fixed in 4% PFA for 24 h, placed in phosphate buffered saline (PBS) for 24 h, transferred to 10% and 20% sucrose for 12–24 h, embedded in optimal cutting temperature (OCT) compound, and frozen. They were cryostat-sectioned longitudinally at 14 µm and mounted on MAS-coated glass slides (Matsunami glass). After washing in PBS for 30 min and PBS with 0.01% Triton X100 (PBST) for 5 min three times, the sections were incubated for 30 min in blocking solution (3% horse serum in PBST) and incubated overnight with the primary antibodies at 4 °C. After washing in PBST for 5 min three times, the secondary antibodies were applied at RT for 1 h. Then, the sections were washed in PBST for 5 min three times and enclosed with 50% glycerol. Anti-Tuj1 mouse monoclonal IgG (1:1000; Covance), anti-Glu-tubulin rabbit polyclonal IgG (1:1000, Millipore), anti-GAP43 rabbit polyclonal IgG (1:1000; Abcam) were also used as primary antibodies. Axons of retinas and optic nerves were observed with the FV1000 confocal microscope (Olympus).

### Axonal tracing

For axonal tracing by anterograde tracer, 3 μL of Biotinylated Dextran Amine (BDA-10,000, Thermo Fisher Scientific) were injected via NanoFiL (World Precision Instruments, Sarasota, FL, USA) into the left eye three days after ONC. Mice were fixed as described above and the left optic nerves were isolated and post-fixed in the same fixative overnight. After washing with PBS, optic nerves were transferred to 10% and 20% sucrose for 12–24 h, embedded in OCT compound, and frozen. They were cryostat-sectioned longitudinally at 14 µm and mounted on MAS-coated glass slides (Matsunami glass). After washing in PBS for 30 min and 0.01% PBST for 5 min three times, the sections were incubated for 30 min in blocking solution and incubated overnight with avidin-Alexa568 at 4 °C. After being mounted with flouromount media, images were obtained with the FV1000 confocal microscope (Olympus). The number of BDA-positive axons extending 0.5, 1, 1.5, 2, 2.5, and 3 mm from the injury site was quantified on at least 4 nerve sections per animals (n = 5). For this purpose, pictures of optic nerve sections were captured as described above. Axons were counted and normalized to the cross-sectional width of the optic nerve at indicated points as described^22^.

### Biochemical analysis

CRMP2 KI and wild-type mice were anesthetized and their optic nerves were removed from the globe to the optic chiasma. In this protocol, three optic nerves made a set. Samples were transferred to tubes, frozen with liquid nitrogen, and kept at −80 °C before use. After measuring their weight using an electronic balance, samples were added to 100 µl of Western lysis buffer, rotated for 1 h at 4 °C, and centrifuged at 1,200 rpm for 15 min at 4 °C. Their supernatants were transferred to another tube, and kept at −20 °C until use. Gene Quaint 1300 was used to measure density. The calibration curve was defined by Bio-Rad protein assay with bovine serum albumin (BSA) solutions. Subsequently, protein concentrations were measured according to Bradford’s method. Moreover, 20 µg/24 µl protein solutions, which consisted of Western lysis buffer, and 4x sample buffer according to densitometry, were boiled for 5 min and applied into a 12.5% polyacrylamide gel. They were subjected to sodium dodecyl sulfate polyacrylamide gel electrophoresis (at 10 mA in stacking gel, at 20 mA in running gel) and their proteins were separated. Proteins in the gel were transferred into an Immoblion®-P Transfer Membrane (for 90 min at 180 mA). Before this, membranes were initialized with 100% methanol and washed with Western blotting buffer. The membranes were incubated with blocking solution for 30 min at 4 °C, incubated with primary antibodies overnight at 4 °C, washed in 0.05% TBST for 10 min twice, incubated with blocking solution, incubated with secondary antibodies, and washed in 0.05% TBST. A signal was detected by enhanced chemiluminescence (ECL) Plus Western Blotting Detection Reagents. The membranes were scanned with a Luminescent Image Analyzer LAS-3000. Primary antibodies used in this study include anti-Tuj1 (Rabbit polyclonal IgG, 1:500, Covance), anti-Tuj1 mouse monoclonal IgG (1:1000; Covance), anti-GAP43 rabbit polyclonal IgG (1:1000; Abcam), and anti- β-actin mouse monoclonal IgG (1:1000; Wako). To quantify the expression levels of the neuron-specific βIII-tubulin (Tuj1) and growth-associated protein 43 (GAP43), expression of β-actin, a housekeeping gene, was measured in the same membrane. The expression of β-actin was divided by the expression of Tuji and GAP43 and a graph was made by the obtained parameter.

### Statistical analysis

All data were graphically presented as the mean ± standard error of the mean (SEM). In case of comparison between two groups, statistical differences were calculated with an unpaired two-tailed Student’s t-test. In case of comparison between three groups, statistical differences were calculated using a one-way analysis of variance (ANOVA) followed by a Tukey’s post-hoc multiple-comparison test as appropriate to the design. GraphPad Prism software version 6.0b was used to perform multivariate statistical analysis.

## Results

### Suppressed depolymerization of microtubules in the optic nerves of CRMP2 KI mice after optic nerve crush

**Figure 1:**
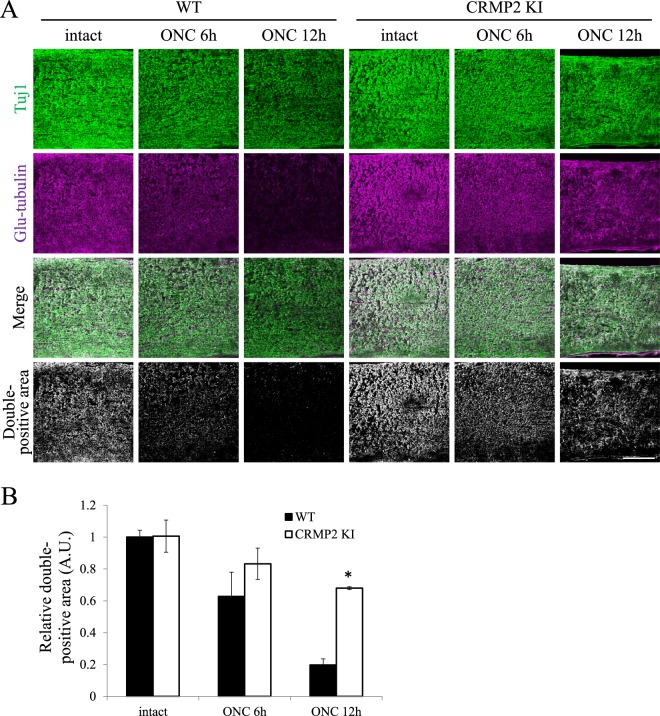
Suppression of microtubule depolymerization in the optic nerves in CRMP2 KI mice. (**A**) Representative images of the double antibody staining for neuron-specific class III β-tubulin (Tuj1, green) and Glu-tubulin (magenta), which are abundant in the polymerized microtubules. The double-positive areas were remarkably reduced after ONC in wild type mice, indicating microtubule depolymerization. However, the double-positive areas were significantly preserved in those from the CRMP2 KI mice. Scale bar: 100 µm. (**B**) Relative quantification of the double-positive areas in the optic nerves of wild type and CRMP2 KI mice at 6 and 12 h after ONC. The data were normalized to the double positive areas on the intact side at 12 h after ONC from the wild type. n = 3 mice for each genotypes. *p < 0.05. Statistical analysis was performed using one-way ANOVA, followed by Tukey’s multi-comparison test. Data are represented as. mean ± SEM. h, hours.

Our previous study on spinal cord injury showed an increased stabilization of microtubules in CRMP2 KI mice after an axonal injury^16^. To analyze the stability of microtubules in the optic nerves, we double-stained the optic nerve sections from wild type and CRMP2 KI mice for the intact side and injured side at 6 and 12 h after optic nerve crush (ONC), with antibodies against neuron-specific class III β-tubulin (Tuj1) and Glu-tubulin that are abundant in polymerized microtubules. In the optic nerves from wild type mice, a decrease in the percentage of double-positive area for Tuj1 and Glu-tubulin was observed as a result of depolymerization of the microtubules after ONC. In contrast, the percentage of double-positive areas was relatively maintained in CRMP2 KI mice at 6 h after ONC compared to WT at 6 hrs ONC and the difference in the double-positive area between wild type and CRMP2 KI mice at 12 h after ONC was statistically significant (Fig. 1). These results indicate a suppressed destabilization of microtubules after ONC in the optic nerves of CRMP2 KI mice.

### Suppression of RGC loss after optic nerve crush in CRMP2 KI mice

**Figure 2:**
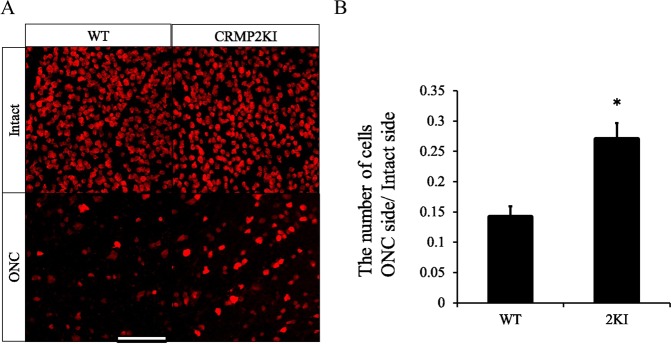
Survival of RGCs 2 wk after ONC in WT and CRMP2 KI mice. Immunostaining of retina from injured and intact sides at 2 wk after optic nerve injury using anti-RBPMS antibody. (**A**) Representative images of RBPMS immunostaining of each side of the retina from wild-type (WT) and CRMP2 KI mice. Scale bar = 100 μm. (**B**) The ratio of numbers of RGC in the injured side against the intact side was calculated. n = 3 mice. *p < 0.05.

Next, we tested whether RGC loss in the retina on the injured side was suppressed in CRMP2 KI mice. To examine this possibility, we used the ONC model by crushing the left optic nerve as described in the method section. Two weeks after ONC, samples of retinas from both sides were subjected to immunostaining with anti-RBPMS to identify the somas of RGCs in the retina (Fig. 2A). The ratio of RGCs in the ONC side to the intact side was calculated and shown as cell viability. Compared to wild-type (14.4 ± 1.5%), cell viability was high (27.3 ± 2.3%) in CRMP2 KI mice (Fig. 2B), suggesting that RGC loss is suppressed in CRMP2 KI mice.

### Degeneration and regeneration of the optical nerve after optic nerve crush in CRMP2 KI mice

**Figure 3:**
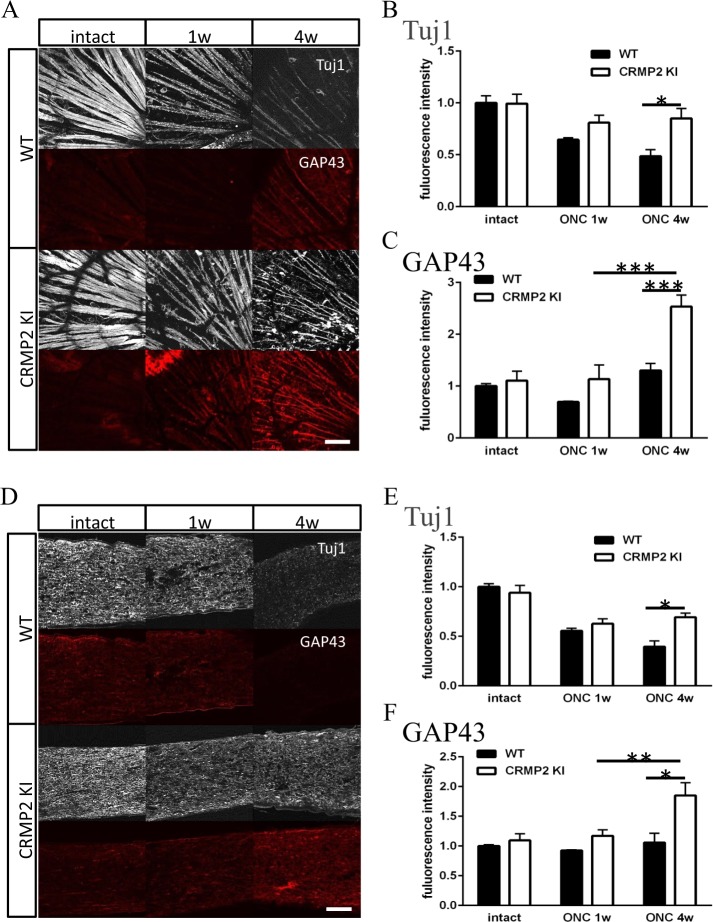
Immunohistochemical analysis of retina and optic nerve after ONC. (**A**) Immunohistochemical images of RGC axon layer of the retina with antibodies for Tuj1 and GAP43. (**B**,**C**) Quantification of Tuj1 (**B**) or GAP43 (**C**) positive area of wild-type (WT) and CRMP2 KI samples after ONC. (n = 4, *p < 0.05 **p < 0.01. Scale bar = 100 µm. (**D**) Images of immunohistochemistry of optic nerve with antibodies for Tuj1 and GAP43 in longitudinal sections. (**E**,**F**) Quantification of Tuj1 (**E**) or GAP43 (**F**) positive area of wild-type and CRMP2 KI samples after ONC. (n = 4 mice, *p < 0.05. Scale bar = 100 µm.

To elucidate axonal stabilization and regeneration, we examined optic nerves from wild-type and CRMP2 KI mice 1 or 4 wk after ONC using immunohistochemistry and western blot analysis. Longitudinal sections of optic nerves were stained with anti-Tuj1 and GAP43 antibody (Fig. 3A). GAP43, a neuron-specific enolase, is used as a molecular marker for axonal regeneration^23^. In wild-type mice, the staining of Tuj1 was gradually decreased (Fig. 3B), and there was almost no change in GAP43 staining (Fig. 3C). On the other hand, in CRMP2 KI mice, the expression of Tuj1 was decreased after ONC, but its decreasing had subsided between 1 to 4 wk after ONC. The expression of GAP43 was not changed 1 wk after ONC, but increased 4 wk after ONC.

To observe RGC axons on the retina after ONC, retinas were immunostained with anti-Tuj1 and GAP43 antibody (Fig. 3D). These antibodies stained bundles of RGC axons and a part of the RGC cell body. The expression of Tuj1 was decreased after ONC in wild-type mice, while there was almost no difference in this expression in CRMP2 KI mice (Fig. 3E). The expression of GAP43 was slightly decreased 1 wk after ONC, but there was almost no change in the wild-type (Fig. 3F). On the other hand, in CRMP2 KI, its expression was markedly increased, which is consistent with the optic nerve data.

**Figure 4:**
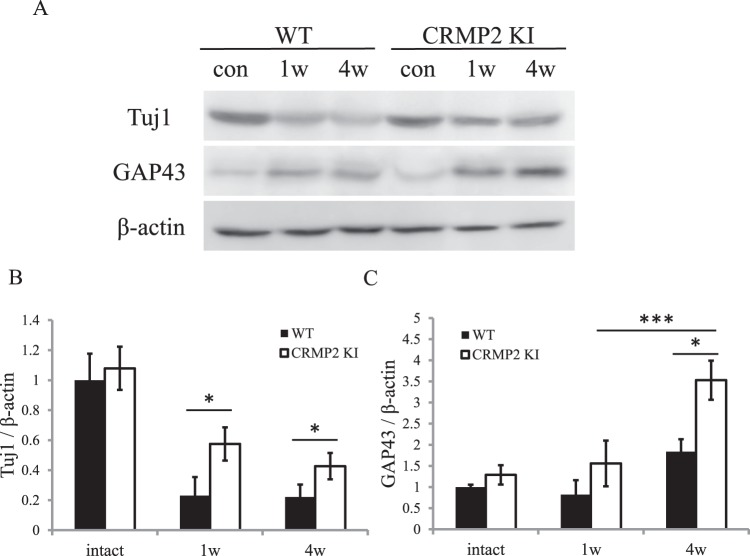
Change in protein levels of Tuj1 and GAP43 in optic nerve after ONC. (**A**) Images of Tuj1, GAP43, and β-actin western blot. (**B**,**C**) Quantification with Tuj1/β-actin (**B**) and GAP43/β-actin. (**C**) n = 4 mice for each genotype, *p < 0.05. Scale bar = 100 µm.

Western blot membranes were blotted with anti-Tuj1, GAP43, and β-β-actin antibody. Expression levels of Tuj1 were dramatically decreased in the wild-type, while its decrease was reduced in CRMP2 KI mice (Fig. 4). In wild-type mice, the levels of GAP43 were slightly increased 4 wk after ONC, but there was almost no difference in levels after ONC. However, in CRMP2 KI, its level was drastically increased 4 wk after ONC (Fig. 4).

**Figure 5:**
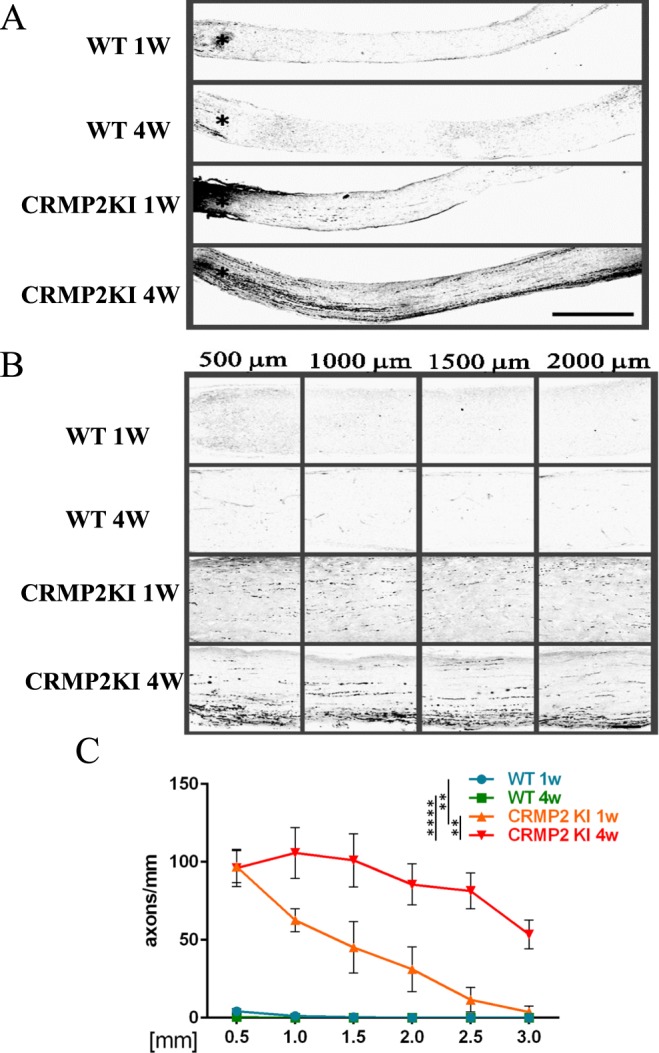
Histological analysis of optic nerve regeneration with anterograde tracer at one and four weeks after ONC in wild-type (WT) and CRMP2 KI mice. (**A**) Longitudinal sections of optic nerves after tracer injection in indicated samples. (**B**) Higher magnification images of the indicated area in (**A**). Scale bar in (**A**) = 500 µm and (**B**) = 100 µm. (**C**) Quantification of regenerating axons at indicated distances beyond the crush site in optic nerves in comparison with WT and CRMP2 KI mice (n = 5 mice for each genotype). **p < 0.01, ****p < 0.0001.

To examine axonal regeneration in the optic nerve after ONC, we injected the anterograde tracer BDA into the left eye and mice were fixed three days later. Longitudinal sections were subjected to histological analysis. As shown in Fig. 5, increased numbers of axons were observed after 1 and 4 wk from ONC in CRMP2 KI mice, indicating the prominent regeneration of optic axons after ONC in CRMP2 KI mice.

## Discussion

The function of CRMP2 is to stabilize microtubules and promote their polymerization, and this function is prevented through the phosphorylation by various kinases including Cdk5, GSK3β and Rho-kinase^24^ Rho-kinase phosphorylates CRMP2 at Thr555^11,25^. In this study, we used CRMP2 KI mice to demonstrate that inhibition of CRMP2 phosphorylation at Ser522 by Cdk5 leads to axonal stabilization and regeneration after ONC. We have shown improved functional recovery after spinal cord injury in CRMP2 KI mice^16^. In spinal cord injury model, we reported relative preservation of stabilized microtubule which is Glu-tubulin positive after injury in CRMP2 KI mice compared to wild type mice^16^. Such differences were also observed in the case of ONC in CRMP2 KI mice (Fig. 1), suggesting un-phosphorylated CRMP2 suppressed depolymerization of microtubule after optic axonal injury. Given this neuroprotective effect, we then analyzed survival of RGC 2 wk after ONC in wild type and CRMP2 KI mice. In CRMP2 KI mice, RGC survival rate 2 wk after ONC also increased (Fig. 2). In CRMP2 KI optic nerves, the presence of Tuj1, a neuron-specific βIII-tubulin, was maintained more than in wild-type ones in the retina and optic nerves (Fig. 3). The presence of GAP43 in distal and proximal axons from the injury site was increased in CRMP2 KI mice (Figs 3 and 4). GAP43 is used as a marker of axonal regeneration. In mature RGC axons, axonal regeneration correlates highly with enhanced expression of GAP43^23,26^. Our experimental results showed an increased presence of GAP43 4 wks after ONC in CRMP2 KI mice (Figs 3 and 4). As the Tuj1 level was maintained after ONC in CRMP2 KI mouse axons, it is possible that axonal regeneration occurs after 4 wk. Our tracing experiments, using the anterograde tracer BDA, showed prominent regeneration of optic axons in CRMP2 KI mice (Fig. 5). These experimental results 1 wk after optic nerve injury are consistent with previous reports regarding the protective effect of the viral introduction of CRMP2T514A into the optic nerve^14,22^. However, our results from analyses 4 wk after ONC may reflect the promotion of axonal regeneration in CRMP2 KI mice (Figs 3–5), suggesting inhibition of CRMP2 phosphorylation suppresses axonal degeneration and promotes axonal regeneration after optic nerve injury. Molecular mechanism we observed in regeneration of optic nerve in CRMP2 KI would be explained by the ability of CRMP2 to promote microtubule polymerization^10^ and actin bundling^27^. Overexpression of CRMP2 promotes axon regeneration of injured motor neurons^28^ and un-phosphorylated CRMP2 retains its function to stabilize microtubule, leading to axon growth in regenerating proximal axons^22,29^.

It is possible for our results to be translated into the development of a therapeutic for patients with optic nerve injury. Previous studies have shown that some molecule microtubule-stabilizing drugs have utility in the regeneration of injured axon^30,31^. The microtubule-stabilizing agent, taxol, and the brain-penetrant microtubule stabilizing agent, epothilone B, have been shown to be effective in axonal elongation after spinal cord injury (SCI)^31^. Epothilone D (EpoD) had improved tau pathology in an AD mouse mode^32^. Lanthionine ketamine (LK) and its synthetic cell penetrating ester derivative, LK ester (LKE) are endogenous sulfur amino acid metabolites^33^. LK directly binds to CRMP2 and alters CRMP2’s protein-protein interactions. LKE administration has been shown to delay progressive neurodegeneration in Alzheimer’s disease (AD) and amyotrophic lateral sclerosis (ALS) mouse models^34,35^. For functional recovery after optic nerve injury, additional treatment to facilitate myelination of regenerated axons will be required because regenerated axons without myelination are not sufficient for behavioral recovery^36^. CRMP2 is also expressed in oligodendrocytes^37,38^ and elevated phosphorylation of CRMP2 at Thr555 by Rho-kinase was observed under oxidative stress and in experimental autoimmune encephalomyelitis (EAE) which is mouse model of human multiple sclerosis^38^. LKE is also beneficial in the outcome of EAE through the suppression of elevated phosphorylation level of CRMP2 at Thr555^39^. These studies suggest that CRMP2 phosphorylation by Rho kinase may play a role in myelination. We recently reported that LKE promoted axonal elongation and functional recovery after spinal cord injury (SCI)^40^. Epothilons and LKE’s therapeutic effects on ONC recovery have not been evaluated, and therefore, it is worth testing these reagents in the ONC model.

In summary, the present study indicates that inhibition of CRMP2 phosphorylation will be a novel approach to the development of treatments for human optic nerve injuries.

## Supplementary information

Supplemental Information
