# Supplementary material for: Genetic inhibition of CRMP2 phosphorylation at serine 522 promotes axonal regeneration after optic nerve injury
Source: Sci Rep. 2019 May 10;9:7188. doi: 10.1038/s41598-019-43658-w (PMC6510754; doi:10.1038/s41598-019-43658-w)

Supplemental Information

Genetic inhibition of CRMP2 phosphorylation promotes axonal regeneration after optic nerve injury

Shunsuke Kondo[1], Kazuya Takahashi[1], Yuki Kinoshita[1], Jun Nagai[1,2], Shuji Wakatsuki[3], Toshiyuki Araki[3], Yoshio Goshima[4], Toshio Ohshima[1, 5, 6]

[1]*Department of Life Science and Medical Bio-Science, Waseda University, Shinjuku-ku, Tokyo, 162-8480 Japan*
[2]*Research Fellow of Japan Society for the Promotion of Science*
[3]*Department of Peripheral Nervous System Research, National Institute of Neuroscience, National Center of Neurology and Psychiatry, 4-11 Ogawa-higashi, Kodaira, Tokyo 187-8502, Japan*
[4]*Department of Molecular Pharmacology and Neurobiology, Yokohama City University Graduate School of Medicine, Yokohama 236-0004 Japan*
[5]*Institute for Advanced Research of Biosystem Dynamics, Waseda Research Institute for Science and Engineering, Waseda University*

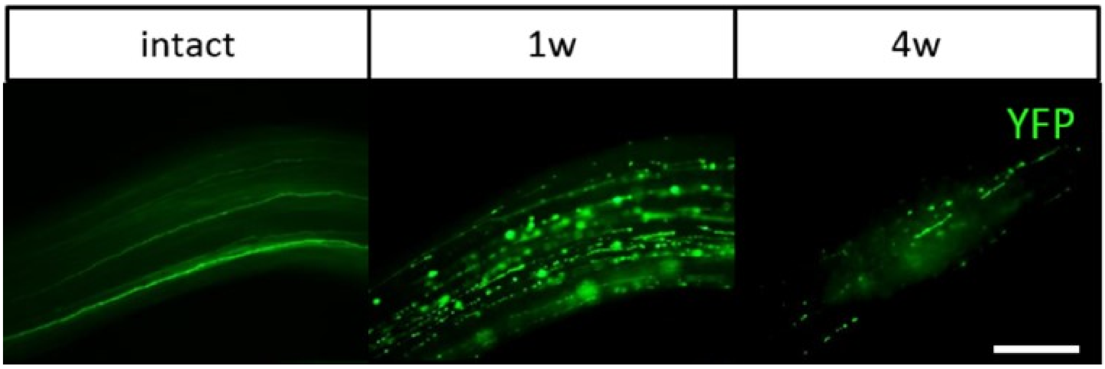

Supplemental Fig.1 Axonal degradation after optic nerve injury in YFP-H mouse
Axonal degradation after optic nerve injury in YFP-H mouse
A small percentage of optic nerve axons are YFP-positive in YFP-H mice (intact). Axonal degradation appeared as the fragmentation of fluorescent signals one week after OCN (1w). YFP-positive axons mostly disappeared four weeks after ONC (4w). Scale bar = 200 μm.

The full scans of Western blotting data are shown in below;

Figure 4 A

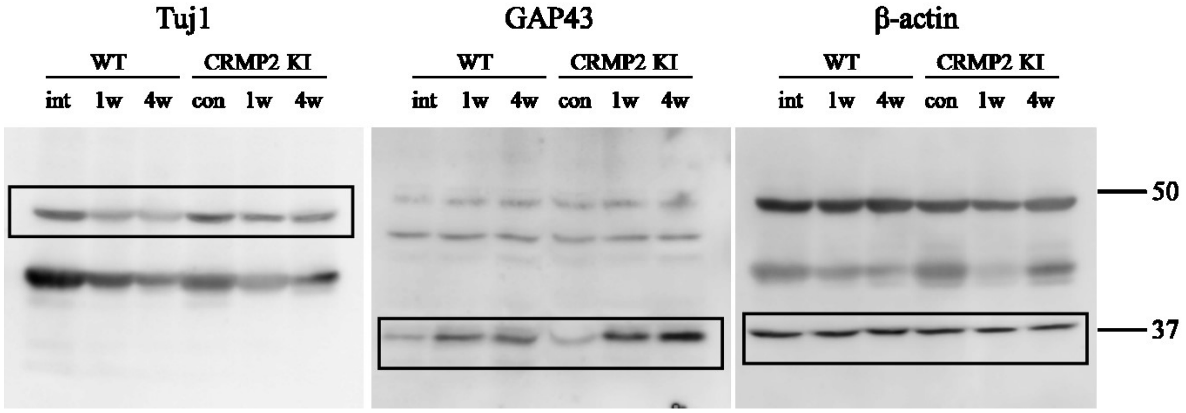

Supplement: Supplementary file 1 — Supplemental Information [file 41598_2019_43658_MOESM1_ESM.pdf]
